# Low-Cost Photolithographic Fabrication of Nanowires and Microfilters for Advanced Bioassay Devices

**DOI:** 10.3390/s150306091

**Published:** 2015-03-12

**Authors:** Nhi M. Doan, Liangliang Qiang, Zhe Li, Santhisagar Vaddiraju, Gregory W. Bishop, James F. Rusling, Fotios Papadimitrakopoulos

**Affiliations:** 1 Nanomaterials Optoelectronics Laboratory, Polymer Program, University of Connecticut, Storrs, CT 06269, USA; E-Mails: nhi.doan@uconn.edu (N.M.D.); liangliang.qiang@gmail.com (L.Q.); zhe.li@uconn.edu (Z.L.); sagar@bio-orasis.com (S.V.); 2 Biorasis Inc., 23 Fellen Road, Storrs, CT 06268, USA; 3 Department of Chemistry, University of Connecticut, Storrs, CT 06269, USA; E-Mails: greg.bishop@uconn.edu (G.W.B.); james.rusling@uconn.edu (J.F.R.); 4 Institute of Materials Science, University of Connecticut, Storrs, CT 06269, USA; 5 Department of Cell Biology, University of Connecticut Health Center, Farmington, CT 062032, USA; 6 School of Chemistry, National University of Ireland, Galway, Ireland

**Keywords:** nanowire, nanogaps, microelectrode arrays, sensor, immunoassay, microfluidic, photolithography, isotropic etching

## Abstract

Integrated microfluidic devices with nanosized array electrodes and microfiltration capabilities can greatly increase sensitivity and enhance automation in immunoassay devices. In this contribution, we utilize the edge-patterning method of thin aluminum (Al) films in order to form nano- to micron-sized gaps. Evaporation of high work-function metals (*i.e*., Au, Ag, *etc.*) on these gaps, followed by Al lift-off, enables the formation of electrical uniform nanowires from low-cost, plastic-based, photomasks. By replacing Al with chromium (Cr), the formation of high resolution, custom-made photomasks that are ideal for low-cost fabrication of a plurality of array devices were realized. To demonstrate the feasibility of such Cr photomasks, SU-8 micro-pillar masters were formed and replicated into PDMS to produce micron-sized filters with 3–4 µm gaps and an aspect ratio of 3. These microfilters were capable of retaining 6 µm beads within a localized site, while allowing solvent flow. The combination of nanowire arrays and micro-pillar filtration opens new perspectives for rapid R&D screening of various microfluidic-based immunoassay geometries, where analyte pre-concentration and highly sensitive, electrochemical detection can be readily co-localized.

## 1. Introduction

**Figure 1:**
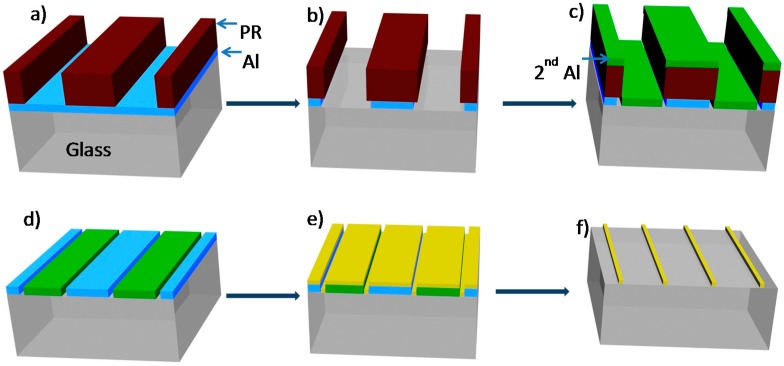
“Edge-patterning” of a thin aluminum film to realize nano- and micron-sized gap and wires. The process starts via photoresist (PR) patterning (**a**); isotropic etch to realize Al undercut (**b**); evaporation of a 2nd Al layer (**c**); followed by photoresist lift-off to realize nano- and micron-sized gaps (**d**); Nano- and micron-sized wires are subsequently formed by depositing a higher work function metal onto the gaps (**e**); followed by aluminum lift-off (**f**).

Integrated microfluidic devices have shown significant promise in lowering cost and increasing automation of immunoassay detection [1,2]. A variety of detection methods including amperometry [3,4,5,6,7,8], electrochemiluminescene [9,10,11,12], field-effect transistor (FET) [13,14,15,16,17,18], optical sensing [16,19,20,21], *etc.* have been incorporated within microfluidic devices. Some microfluidic immunoassay devices, have been also outfitted with online micro-filters to pre-concentrate the antigen of interest [19,22,23] or filter out blood platelets [24]. Typical microfilters are composed of large aspect-ratio trenches or micropillars with sub-micron gaps [22,25,26]. Microfilters have been fabricated by a number of sophisticated techniques involving electron-beam-lithography [27,28,29], deep-reactive ion etching [22], nanoimprinting [28,30], and dip-pen nanolithography [31]. The inherent expenses associated with these fabrication methodologies naturally inflate R&D costs, where multiple iterations are needed to optimize a certain device configuration. The ingenious “edge patterning” technique of Whitesides *et al*. [32], have enabled the formation of nano- to sub-micron sized features using traditional photolithography and isotropic etching of a reactive metal (*i.e.*, aluminum). As shown in Figure 1, metal undercutting beneath the photoresist edge results in nanosized trenches with widths of *ca.* 50 nm. The widths of these trenches are typically limited by the grain-size inhomogeneity of the deposited metal film [32].

In this contribution, we describe the use of edge-patterning method to produce nanowire-based array electrodes along with high-aspect ratio microfilters that can be both integrated within PDMS (polydimethylsiloxane)-based microfluidics. The latter approach enables selective localization and pre-concentration of micron-sized beads at predetermined locations, which is important for electrochemical-based immunodetection [23]. In this manner, a number of signal amplification techniques can be implemented, as a consequence of the high surface-to-volume ratio that these microbeads provide [8,22,23,33,34,35], alongside the facile incorporation of large number of enzyme labels, (e.g., horseradish peroxidase, glucose oxidase, *etc.*) compared to capturing antibodies [8,36]. While magnetic microbeads can be easily localized using magnetic fields, micropillar-based filters extend such localization to non-magnetic beads as well. With a typical size of microbead diameter between 1 and 10 µm, fabrication of high-aspect ratio micropillar filters using traditional photo-lithography is a challenging task that requires the use of fairly expensive, high-end photolithography masks [25]. We herein show that high resolution, custom-made masks can also be readily fabricated using the edge-patterning method. These are based on inexpensive plastic masks in combination with traditional UV photolithography or a handheld UV lamp to achieve gaps of *ca.* 4 microns and height-to-width aspect ratio of 3. Additionally the as-produced edge-patterned gaps can be used to define micron- to nano-sized wires for microelectrode arrays [37]. Such microelectrode arrays can significantly amplify electrochemical signals due to the radial mass transport of electroactive species towards the sensor electrode [38]. The use of edge-patterning method to co-localize nanowire arrays and micro-pillar filtration opens new capabilities for the facile production of advanced microfluidic devices that could ultimately lower the cost and increase automation for electrochemical immunoassays [39].

## 2. Experimental Section

### 2.1. Materials and Instrumentation

All chemicals were procured from Aldrich (St. Louis, MO, USA). Gold (99.99%), silver (99.99%), aluminum (99.99%), and chromium (99.99%) were obtained from Kurt Lesker (Jefferson Hills, PA, USA). All glass substrates were Fisherfinest microscope slides from premium plain glass. Shipley *S1813* photoresist, Developer-351, SU-8 3010, and SU-8 developer were purchased from Microchem Corp. (Newton, MA, USA). Sylgard 184 PDMS pre-polymer and corresponding curing agents were obtained from Dow Corning (Midland, MI, USA). Photolithography was carried out using a MA-6 mask aligner (Karl Süss, Sunnyvale, CA, USA). Scanning electron microscopic (SEM) images were collected by a JSM-6335F instrument (JEOL, Peabody, MA, USA) with a cold cathode field emission source at 10.0 kV and working distance of 8 mm. 

### 2.2. Nanogap Formation

Nanowires and nanogaps were constructed on glass substrates, which were sequentially cleaned by sonicating in aqueous suspension of Micro-90 soap, then acetone, and then deionized (DI) water, for 30 min each. Following 4 min oxygen plasma treatment of these substrates (Plasma Gas System 210, PVA TePla America, Corona, CA, USA, Oxygen flow at 220 sccm, pressure 800 mTorr, 400 W), thermal evaporation of Al (or Cr) metal (*ca*. 100 nm) was conducted on a Denton Vacuum (Moorestown, NJ, USA) DV 502A evaporator at a base pressure of 10^−6^ Torr and an average deposition rate of 10 Å/s. Subsequently, 1.5 μm of S1813 photoresist was spin-coated and baked at 120 °C for 10 min. Following UV exposure, development and stripping (using oxygen plasma), the exposed metal was over-etched in a mixture of H_3_PO_4_:H_2_O:HNO_3_:CH_3_COOH (16:2:1:1 *v*/*v*). Prior to photoresist removal, a second layer of Al was thermally deposited. Subsequently, the substrate was sonicated in acetone for 30 min to facilitate photoresist lift-off, followed by another oxygen plasma cleaning step to remove adsorbed organic residue from the Al nanogaps.

### 2.3. Nanowire Fabrication

Glass substrates with Al nanogaps (described above) were loaded into a high vacuum evaporator and 20 nm of Cr film was thermally evaporated at deposition rate of 10 Å/s. Subsequently, the aluminum layer was etched away (lifted off) after 2 min exposure to a 1 M aqueous KOH solution, resulting Cr nanowires. For gold and silver nanowires, a thin layer of chromium (5 nm) was first deposited, followed by deposition of 40 nm gold or silver at a deposition rate of 10 and 20 Å/s.

### 2.4. Micropillar-Based Microfluidic Photomask Fabrication

Three hundred (300) nm thermally evaporated Cr film was first deposited on glass substrates at a rate of 10 Å/s. Subsequently, 1.5 μm of S1813 photoresist was spin-coated on top of the Cr film and baked at 120 °C for 10 min. Following UV exposure, development and stripping (using oxygen plasma), the exposed metal was over-etched for 15 min using a Cr etchant solution obtained from Sigma Aldrich composed of diammonium hexanitratocerate (10%–30%) and nitric acid (5%–10%)). Following an aqueous wash and an oxygen plasma cleaning steps, an additional 300 nm thermally evaporated Cr film was deposited at a rate of 10 Å/s. Subsequently, the S1813 photoresist was lifted off by sonicating in acetone for 30 min followed by another oxygen plasma cleaning step. The resulting microgap pattern was then adjusted to the desired length using another mask (defined as cutting mask). For this the microgap length was defined with a second photolithography step using S1813 photoresist that was spin-coated and pre-heated at 120 °C for 10 min (producing a average thickness of 1.5 µm) before exposing through a “cutting mask” that is placed perpendicular to the original microgap pattern. After UV exposure, photoresist development and 90 s Cr etch, the unexposed photoresist was stripped in acetone and cleaned in DI water and oxygen plasma. Subsequently, the resulting pattern was incorporated into the microfluidic channel using a third photolithographic step, similar as above (*i.e.*, spin-coating of S1813 photoresist, UV-exposure, development and 300 nm thermal evaporation of Cr) before sonication-assisted lift-off in acetone for 30 min.

### 2.5. Microfluidics Device Fabrication and Microbead Filtration

A clean silicon (Si) wafer was spin-coated with 12 µm-thick of SU-8 3010. After pre-baking for 1 min at 65 °C and 12 min at 95 °C, the SU-8 3010 film was exposed using the Cr photomask realized in Section 2.4. Following a 12 min post-bake step at 95 °C, the SU-8 3010 was developed and further hard baked at 150 °C for 90 min. Subsequently, PDMS pre-polymer and its corresponding curing agent were mixed at a 10:1 volume ratio and casted over the SU-8 mold. After curing at 90 °C for 90 min, PDMS was peeled off the mold and two access holes where punched out, via a blunt-end 23-gauge needle, to serve as liquid inlets and outlets. Both PDMS mold and glass slides were then washed with soap, rinsed thoroughly, air-dried, exposed to oxygen plasma (4 min), affixed together and then heated at 90 °C for 1 h. Fluids were delivered and withdrawn from microfluidic devices using polyethylene (PE) tubes with inner diameter 0.28 mm that were inserted into the pre-punched inlet and outlet holes. The desired fluid flow was established using a syringe pump, connected to the outlet and the microfluidic device and operated in withdraw mode.

## 3. Results and Discussion

Recent advances in high-resolution inkjet and laser printing have enabled the facile production of plastic masks at a fraction of the cost of high-end chrome analogues. The only drawback of plastic masks is that the smallest feature clearly definable is 25 microns. By extending the “edge-patterning” method of Whitesides *et al.* [32] we herein investigate the ability to use plastic masks for the formation of: (i) nanowires; (ii) sub-micron photo masks; and (iii) high-aspect ratio micropillar filters.

### 3.1. Nanogaps and Nanowires

Figure 1 illustrates the fabrication of nanogaps and nanowires based on “edge-patterning” of a thin aluminum film. The process starts with deposition of 100 nm Al via thermal evaporation or other deposition techniques on a substrate. The choice of substrates can vary widely from glass, silicon or other insulating, semiconducting or metallic substrates, whose work functions are substantially higher than that of Al. Subsequently, a thick positive photoresist layer (*i.e.*, 1.8 µm) was spin-coated onto the Al layer and patterned via UV lithography through a plastic photomask. After photoresist development, (Figure 1a) the exposed Al pattern was dissolved using a 16:2:1:1 *v*/*v* mixture of H_3_PO_4_:H_2_O:HNO_3_:CH_3_COOH. This ratio allows for slow Al etching that not only dissolves the exposed metal but also gradually etches the aluminum underneath the photoresist (Figure 1b). As described below, the width of the Al undercutting at the photoresist edge can be controlled depending on the exposure time to the aluminum etchant. Subsequent deposition of a second Al layer (green, Figure 1c), followed by acetone-assisted photoresist lift-off (Figure 1d) produces a gap, that depending on etching time and to a lesser extent on the granular size of Al, it can vary from nanometer size to several microns. Such gaps have the exact shape of the photoresist edge that was originally patterned on the substrate (Figure 1a).

**Figure 2:**
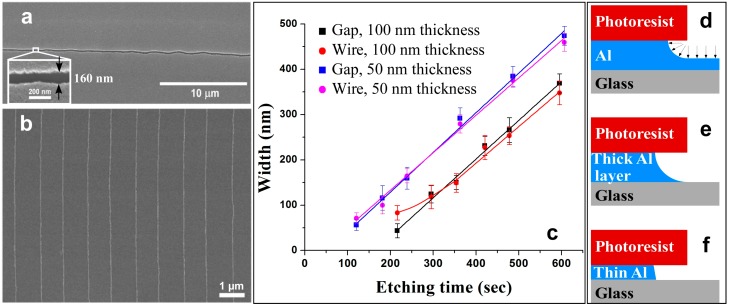
(**a**) SEM images of Al nanogaps formed using the process described in Figure 1a–d; (**b**) Representative Cr nanowires realized by thermal evaporation of chromium onto the Al-nanogaps of (**a**) and Al lift-off (shown in Figure 1e,f); (**c**) Nanogap and nanowire width for two Al thickness layers, as a function of Al etching time. The Al undercut produced by isotropic etching (**d**) works less optimally for thick (**e**); as opposed to thin (**f**) Al layers and small etching times.

Figure 2a demonstrates typical Al nanogaps realized by edge patterning method. Nanogaps with width of *ca.* 160 ± 13 nm are obtained by exposing a thin (50 nm) Al layer for 175 s to the aforementioned H_3_PO_4_:H_2_O:HNO_3_:CH_3_COOH etchant mixture. Such nanogaps are relatively uniform and continuous along the entire perimeter of the photolithographed object. The resulting Al nanogap can then be used as a lift-off layer to pattern various types of nanowires (NWs) as illustrated in Figure 1e,f. For this, a variety of metals (*i.e.*, Au, Ag, Pd, Cr, Ti, Ni, *etc.*) and semiconductors (*i.e.*, CdSe, CdS, TiO_2_, ZnO, In_2_O_3_ and SnO_2_ *etc.*) can be deposited via thermal evaporation, sputtering, e-beam or chemical vapor deposition (CVD) followed by an Al lift-off step. Such Al lift-off (Figure 1f) is realized in either acidic (*i.e.*, H_3_PO_4_:H_2_O:HNO_3_:CH_3_COOH etchant mixture) or basic (1 M KOH) media, depending on the compatibility of metal or metal oxide based nanowires and etching solution. When compared to lithographically patterned nanowire electro-deposition (LPNE) method that was reported by Penner *et al.* [37,40], the edge-patterning method described herein offer a highly flexible venue to realize nanowire architectures. Moreover, unlike LPNE that requires conductive surfaces and environmental unfriendly metal precursors, Al lift-off is compatible with all substrates and common acids and bases.

To estimate the minimum width of nanowires that Al lift-off process can produce, the effect of etching time was studied. Figure 2c illustrates both nanogap and nanowire widths as a function of etching time for two Al layer thicknesses (*i.e.*, 100 and 50 nm). Similar etching rates (*ca.* 0.91 nm/s) were obtained for both thick and thin Al layers, indicating that the rate limiting step is 1-D diffusion of etching reagents and soluble byproducts across the undercut. In general, a near 1-to-1 correspondence is observed between the nanogap width and nanowire diameter [37]. For thick Al layers and small etching times, however, the system deviates from good replication. For example, when the nanogap approaches the thickness of the Al layer, the nanogaps show a significantly rougher edge and large number of discontinuities (not shown), with the nanowire significantly larger (*i.e.*, (85 ± 16 nm) than that of the gap (*i.e.*, 42 ± 15 nm). This is due to the nature of isotropic wet etching (Figure 2d), which leaves a thin Al layer on glass surface and is also responsible for: (a) increasing the average nanowire thickness (shown by the departure of red curve from the black curve in Figure 2c, and (b) decreasing the adhesion between the nanowire and glass surface. Correspondingly, the profile of the thin Al layer (*i.e.*, 50 nm) becomes much sharper than that of thick Al layer (*i.e.*, 100 nm), as shown in Figures 2c,e,f [41]. Using 50 nm thin layers, continuous nanogap and nanowires were realized around various patterns. Typically, the thinnest continuous nanogap obtained was *ca.* 50 ± 10 nm, while the corresponding nanowire was slightly larger at *ca.* 70 ± 20 nm (Figure 2c).

**Figure 3:**
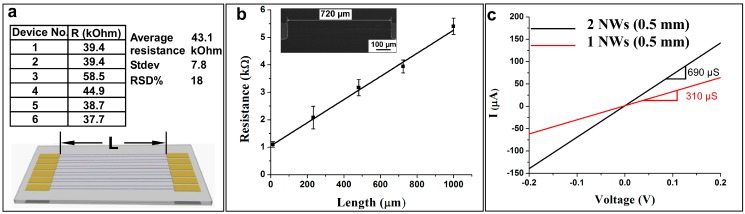
(**a**) Six pairs of silver nanowires with Cr contact pads at their ends. Insert table shows the electrical resistance values of six pairs of 43 nm thick, 400 nm wide and 2 cm (20,000 µm) long Ag nanowires; (**b**) 2-point probe electrical resistance of a Ag nanowire (with 43 nm in thickness and 300 nm in width) as a function of nanowire length. Inset in (b) shows the SEM image of a 720 µm long nanowire with its Cr contact pads; (**c**) I-V characteristics of one (red) and two (black) Ag nanowire(s) with 43 nm in thickness, 300 nm in width and 500 µm in length.

To demonstrate the continuity of these nanowires over large distances (up to 20 mm in length), we tested their current-voltage (I-V) characteristics using a simple two-point-probe method. For this, two Cr contacting pads were patterned at the ends of the nanowires (Figure 3a) via spin-coating positive photoresist and using another plastic mask to photolithographically pattern contacts at the desired nanowire length (L) (Figure 3a). Figure 3b illustrates that the electrical resistance of Ag nanowires with 43 ± 2 nm in thickness and 300 nm in width varies linearly with their length (L) (*i.e.*, from 12 to 1000 μm). This indicates the structure uniformity of these nanowires over long lengths, with no significant defects presented. To demonstrate the uniformity that the Al lift off approach affords in making these nanowires, Cr contacts were deposited onto two 43 by 300 nm nanowires with that were 500 µm spaced apart. As shown in Figure 3c, the conductance of both nanowires can be measured first and subsequently, one nanowire can be selectively severed with a razor blade scratch. The conductance of two 500 µm long nanowires is 690 μS, while the single nanowire conductance was nearly half (*i.e*., 310 μS), which is within 10% error. In order to get a better estimate of nanowire uniformity, the resistivity of six pairs of 43 by 400 nm Ag nanowires with length of 2 cm (20,000 µm) (Figure 3a) were measured using 2-point probe. The insert table in Figure 3a shows that the resistance of the six nanowire pairs is fairly uniform, averaging 43 kΩ and with standard deviation of 18%. Both aforementioned long and short nanowire resistivity tests indicate that the Al lift-off method is capable of preparing continuous, defect-free nanowires with good electrical uniformity, that can easily extend from a couple to tens of thousands of microns in length. 

### 3.2. Micropillar-Based Filtration within Microfluidic Devices

The low cost and high fidelity of the Al lift-off method described above can not only be used to realize nanowire arrays but also assist in incorporating micropillar-based filtration within microfluidic devices. Such micropillar-filtration could be designed for on-line separation of large objects (*i.e.*, microspheres, cells, aggregates, *etc.*) within low-cost microfluidic devices for point-of-care diagnostics. With this in mind, we tested the applicability of the “edge-patterning” method [32] in assisting to localize 6 µm of beads within a confined area of a microfluidic device.

**Figure 4:**
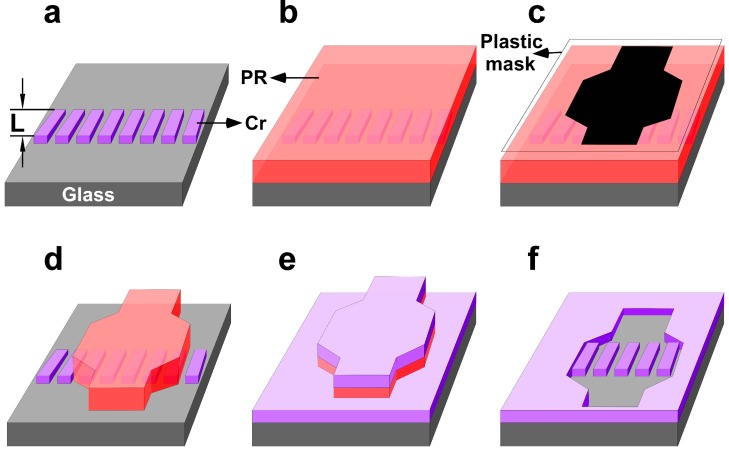
Incorporation of the array of microgaps (**a**) into a photomask to produce microfiltration masters for PDMS microfluidics. Following positive photoresit spin coating (**b**), photo-patterning with a plastic mask that defines the microfluidic channel (**c**) protects the microgaps (**d**) from Cr filling (**e**) until lift-off (**f**), which completes the formation of a high resolution, homemade, microfiltration photo-mask.

Figure 4 illustrates the key steps to incorporate micron- (and ultimately sub-micron) gaps within a mask that can be used to fabricate the master (mold) for PDMS-based microfluidic channel. For this, Cr is substituted for the Al layer of Figure 1a, and used in “edge-patterning” to produce micron-sized gaps. These Cr gaps allow the formation of cheap, high-resolution photomasks for optical patterning of continuous stripes with edge-fidelity comparable to high-end photomasks [42,43]. For this, 300 nm thermally evaporated Cr film was deposited on a glass substrate. After positive photoresist patterning using a plastic mask with rectangular 25 by 500 µm features that were spaced apart by 25 µm gaps, the Cr was etched using a commercial diammonium-hexanitratocerate/nitric acid mixture (see experimental) for 15 min to produce a 2 µm undercut beneath the photoresist (Figure 1b). Subsequently, another 300 nm thermally evaporated Cr film was deposited (Figure 1c and lifted off (Figure 1d) to produce a continuous 2 µm gaps around the edge of the rectangular 25 by 1000 µm features. In order to transform these rectangular Cr gaps into an array of Cr lines shown in Figure 4a, their tops and bottoms together as well as their surrounding Cr must be removed. This was realized by patterning positive photoresist across the rectangular Cr gaps with a width (L) as shown in both Figure 3a and Figure 4a. Following Cr etch and photoresist strip-off, an array of 2 µm Cr gaps is realized on top of glass substrate (Figure 4a). Figure 4b–f illustrate the subsequent steps in order to insert such array of 2 µm Cr gaps within a much larger microfluidic channel. In brief, positive photoresist was spun on top of the Cr gap array (Figure 4b) and then roughly aligned with of microfluidic channel plastic photomask (Figure 4c). Following UV-exposure and photoresist development (Figure 4d) a third layer of Cr (300 nm) is thermally evaporated (Figure 4e) and lifted off to produce a homemade micropillar filter incorporated within a microfluidic channel. In such fashion, the size, shape and length of the microgaps can be readily adjusted in order to vary and eventual optimize the performance of the microfluidic filtration device described below.

**Figure 5:**
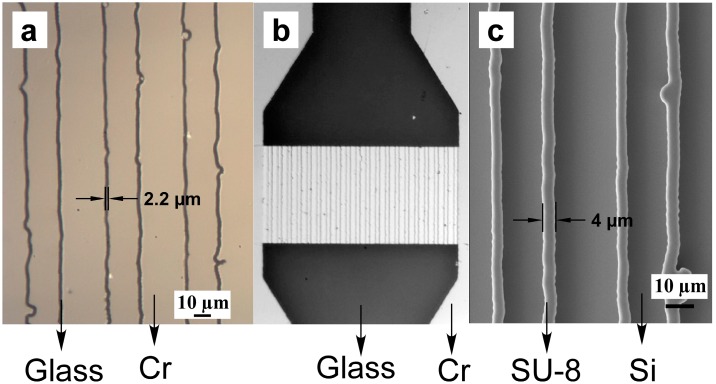
Optical and SEM micrographs of the homemade Cr mask utilized to form an array of Cr microgaps (**a**) within a microfluidic channel (**b**). This mask is then used to photolithographically pattern SU-8 photoresist in order to realize an array of rectangular-shaped micro-pillars (**c**).

Figure 5 illustrates optical and SEM micrographs of the high resolution, custom-made, microfiltration photo-mask, whose fabrication is described above. As shown in Figure 5a, 2.2 µm continuous Cr gaps are realized, despite the edge irregularities and roughness of a lesser quality, rectangular starting plastic photomask. Despite the roughness and irregularities the edge-patterning method affords the formation of continuous channels devoid of any blockage. Figure 5b depicts the incorporation of the array of Cr gaps within a microfluidic channel (*i.e.*, top and bottow black gaps). The widened filtration region with respect to the inlet and outlet (shown on top of Figure 5b) of the regular microfluidic channel is designed in order to account for the flow resistance through the microfiltration array.

The homemade mask (Figure 5b) was then used to realize masters for the production of PDMS microfluidics. For this a negative SU-8 3010 photoresist was spin coated on Si wafer with thickness of *ca.* 10 µm after 1 and 10 min pre-baking at 65 and 95 °C, respectively. Following 0.8 s UV exposure (with UV light intensity *ca.* 30 mW/cm^2^) and 12 min post-bake at 95 °C, the substrate was soaked in SU8 developer for 6 min and washed thoroughly with additional SU8 developer and isopropanol before being dried with air. Figure 5c depicts an SEM micrograph of the resulting SU8 rectangular micropillars with an average width and height of 4.0 and 10 µm, respectively (*i.e.*, cross-sectional aspect ratio of 2.5). Such micropillars are continuous and closely trace the edge roughness of the homemade Cr mask. The nearly double width of SU-8 micropillars compared to that of the gaps of the homemade Cr mask is attributed to edge diffraction effects [44,45] The use of SU-8 3010 as opposed to SU-8 2010 and others was chosen because of its reported higher adhesion to Si wafers. This is important to ensure the continuity of SU-8 micropillars and their resistance against delaminating from their Si substrates following the PDMS removal (described below).

**Figure 6:**
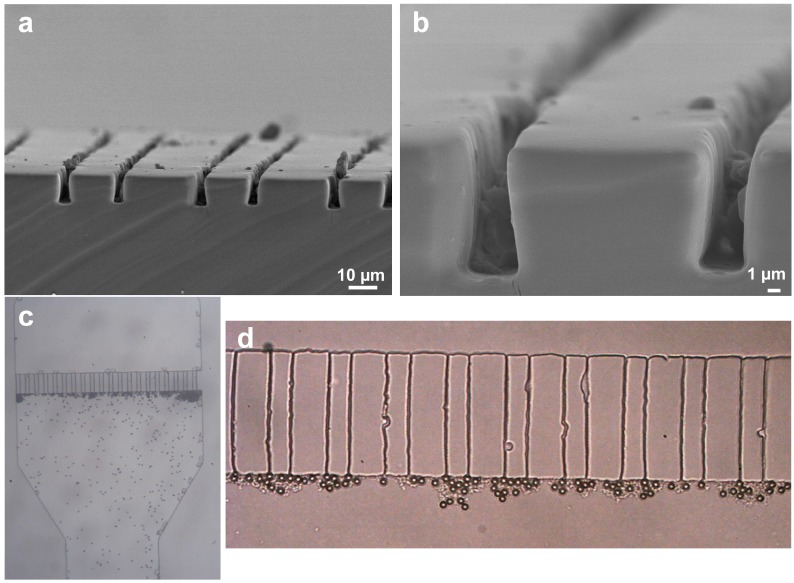
PDMS microfluidic devices that incorporate micropillar-based filters, obtained from homemade Cr-made mask and SU-8 3010 masters shown in Figure 5. (**a**) and (**b**) illustrate SEM cross-sections of the PDMS micropillar filters. (**c**) and (**d**) show optical micrographs of PDMS-incorporated micropillar filters to arrest the flow of 6 µm beads, while allowing the rest of the solution to flow through.

The targeted microfluidic device was fabricated by casting the mixture of PDMS prepolymer and crosslinking agent over the aforementioned SU-8 3010 micropillars. Following vacuum treatment to remove all dissolved air and bubbles, the mixture is heated on a hot plate at 90 °C for 2 h, allowed to cool down at room temperature and then peeled-off. Figure 6a,b illustrate the cross-sectional view of the micropillar gaps. The bottom width of micropillar gaps closely tracks the SU-8 3010 micropillar master. At the other end, the gap shrinks by *ca.* 25% to 3 µm, as shown clearly in Figure 6b. Such unexpected size reduction, is attributed to the elastic nature of PDMS that improves the cross sectional aspect ratio from 2.5 (of the master) to 3 (for the mold). Following needle punching of an inlet and outlet at each end of the microfluidic channel, the PDMS was plasma-treated together with the glass slide to which it was later affixed by heating at 90 °C for 1 h to afford an irreversible bond between the two [46]. Subsequently, a dilute mixture of 5.8 µm microbeads with bead concentration ranging from 0.001 mg/mL to 5 mg/mL in phosphate buffer saline (PBS) was introduced into the microfluidic channel using a needle-assisted suction from the other end. As shown in Figure 6c,d, as well as the video in the Supplementary Information section, the aforementioned micropillar gap device can effectively stop the passage of these microbeads, without blocking the fluid flow. This is attributed to the fact that the channel height cannot tolerate two microbeads on top of each other (otherwise the 2nd layer would have come in focus in the video, when intentionally defocusing away of the 1st layer), therefore allowing the uninterrupted flow of PBS, while no microbeads managed to traverse the micropillar gap barrier. This demonstrates the viability of the micropillar gap filtration method using low-cost home-made masks with traditional UV-photolithography, thereby negating the need of expensive commercial masks [25] and expensive deep-reactive ion etch (DRIE) masters [22].

### 3.3. Conclusions and Outlook

The work described expands on the use of edge-patterning [32] based on isotropic wet etching underneath a patterned photoresist to define nano- to micron-sized gaps, wires and high-aspect ratio pillars. The height and width of these features depend on the thickness of the sacrificial low-work function metal (*i.e.*, Al) and etching duration, respectively. Using the Al edge-patterning method, the formation of continuous and highly uniform Cr nanowire arrays was demonstrated with height and thicknesses of few tens and hundreds of nm, respectively, while their length spans to cm in size and electrical uniformity ranging from 10% to 20%. Such patterning can be extended to a variety of high work function metals and semiconductors and further enrich nanowire architectures. Moreover, we show that edge-patterning can be translated from Al to Cr, thereby enabling its use for the formation of high resolution, custom-made Cr masks which are constructed from relatively inexpensive plastic masks. These custom-made Cr masks are ideal for patterning high-aspect ratio features for electronic, photonic, and biosensor based applications. Using this concept, we produced micro-pillar SU-8 masters and corresponding PDMS microfluidic molds with 3–4 µm gaps, suitable for filtering out 6 µm beads. The ability to spatially define nanowire arrays and microgap filters may offer a large advantage for automated microfluidic electrochemical immunoassays where microelectrode 3D diffusion and sample pre-concentration can play a crucial role for signal amplification. In general, the approach described herein may facilitate the integration of microfluidic devices with a variety of nano/micro concepts to improve detection limits and lower costs for the eventual development of cheap point-of-care medical diagnostics based on detection of molecular biomarkers in human body fluids.

## Supplemental Information

A micro-filtration video illustrating that the PDMS micropillar array is capable to retain and concentrate a 2.5 mg/mL dispersion of 6 µm micro-beads, while the suspending fluid (10 mM PBS) flows smoothly through.

## Supplementary Files

Supplementary File 1
